# Institutional Life at Its Best

**Published:** 1911-10-07

**Authors:** 


### INSTITUTIONAL LIFE AT ITS BEST.

We said in 1886, to the surprise of many, that Lowell's noble words well describe the spirit of the best workers in our hospitals, asylums, and other institutions for the cure and relief of the suffering. In the years which have elapsed since these words were written, the privilege of personal service in the cause of the sick has become, by deliberate choice, a spirit of action in the daily life of thousands of our countrymen and countrywomen. Many of these not otherwise working in the hospital field, from the King downwards, systematically devote a portion of their time every year to help institutions for the sick. It is not possible nowadays to attend a gathering of institutional workers without being struck by the conviction that these services for the sick and disabled are regarded as a privilege, and that the work so done is in fact precious and inspiriting to the workers. For the institutions, and for the managers also, no better stimulus could be desired or imagined. To soothe the sufferers, to restore the injured, to comfort the dying, and to cure the sick must at all times be a God-like work. If this be true, then the wider such influences are extended, the better for the nation and the people at large. And between the parish or congregation, the family or household, and even the individual and the hospital and its workers, there is that touch of nature, that golden chord of human sympathy, which makes the whole world kin. Every parish, congregation, family, household, and individual must encounter the dark days of human weakness, and must one day realise disease in their own persons, or in the persons of those who are nearest and dearest to them.

